# Moderate traumatic brain injury, acute phase course and deviations in physiological variables: an observational study

**DOI:** 10.1186/s13049-016-0269-5

**Published:** 2016-05-23

**Authors:** Stine B. Lund, Kari H. Gjeilo, Kent G. Moen, Kari Schirmer-Mikalsen, Toril Skandsen, Anne Vik

**Affiliations:** Department of Neurosurgery, St. Olavs Hospital, Trondheim University Hospital, Trondheim, Norway; Department of Cardiothoracic Surgery, St. Olavs Hospital, Trondheim University Hospital, Trondheim, Norway; Department of Medical Imaging, St. Olavs Hospital, Trondheim University Hospital, Trondheim, Norway; Department of Anaesthesia and Intensive care Medicine, St. Olavs Hospital, Trondheim University Hospital, Trondheim, Norway; Department of Physical Medicine and Rehabilitation, St. Olavs Hospital, Trondheim University Hospital, Trondheim, Norway; Department of Circulation and Medical Imaging, Faculty of Medicine, NTNU, Norwegian University of Science and Technology, Trondheim, Norway; Department of Neuroscience, Faculty of Medicine, NTNU, Norwegian University of Science and Technology, 3250 Sluppen, N-7006 Trondheim, Norway; Department of Nursing Science, Faculty of Health and Social Science, NTNU, Norwegian University of Science and Technology, Trondheim, Norway

**Keywords:** Moderate traumatic brain injury, TBI, Physiological variables, Guidelines, Acute phase, Deviations

## Abstract

**Background:**

Patients with moderate traumatic brain injury (TBI) are a heterogeneous group with great variability in clinical course. Guidelines for monitoring and level of care in the acute phase are lacking. The main aim of this observational study was to describe injury severity and the acute phase course during the first three days post-injury in a cohort of patients with moderate TBI. Deviations from defined parameters in selected physiological variables were also studied, based on guidelines for severe TBI during the same period.

**Methods:**

During a 5-year period (2004–2009), 119 patients ≥16 years (median age 47 years, range 16–92) with moderate TBI according to the Head Injury Severity Scale were admitted to a Norwegian level 1 trauma centre. Injury-related and acute phase data were collected prospectively. Deviations in six physiological variables were collected retrospectively.

**Results:**

Eighty-six percent of the patients had intracranial pathology on CT scan and 61 % had extracranial injuries. Eighty-four percent of all patients were admitted to intensive care units (ICUs) the first day, and 51 % stayed in ICUs ≥3 days. Patients staying in ICUs ≥3 days had lower median Glasgow Coma Scale score; 12 (range 9–15) versus 13 (range 9–15, *P* = 0.003) and more often extracranial injuries (77 % versus 42 %, *P* = 0.001) than patients staying in ICU 0–2 days. Most patients staying in ICUs ≥3 days had at least one episode of hypotension (53 %), hypoxia (57 %), hyperthermia (59 %), anaemia (56 %) and hyperglycaemia (65 %), and the proportion of anaemia related to number of measurements was high (33 %).

**Conclusion:**

Most of the moderate TBI patients stayed in an ICU the first day, and half of them stayed in ICUs ≥3 days due to not only intracranial, but also extracranial injuries. Deviations in physiological variables were often seen in this latter group of patients. Lack of guidelines for patients with moderate TBI may leave these deviations uncorrected*.* We propose that in future research of moderate TBI, patients might be differentiated with regard to their need for monitoring and level of care the first few days post-injury. This could contribute to improvement of acute phase management.

## Background

Patients with moderate traumatic brain injury (TBI) have great variability in injury severity and acute phase course. They may have both intra- and extracranial injuries possibly inducing secondary brain injury during the acute phase [1–5]. Nevertheless, recommendations for acute phase treatment and monitoring of physiological variables are lacking [4–8]. Several studies in patients with severe TBI have demonstrated that secondary insults, such as deviations in physiological variables, are associated with adverse outcome [9–16]. Thus, implementation of guidelines [17, 18] for observation and treatment in intensive care units (ICUs) to avoid secondary brain injury have improved outcome in patients with severe TBI during the last decades [17, 19]. On the other hand, contemporary studies describing injury severity and the acute phase in moderate TBI are scarce [3, 4, 7, 8], which makes it difficult to evaluate and improve acute phase management for this patient group [1]. Also, results in studies of patients with moderate TBI are often reported together with results of patients with severe TBI, making precise judgments about this particular group difficult [6–8, 20].

The main aim of this observational study was to describe injury severity and the acute phase course the first three days post-injury in a cohort of patients with moderate TBI at a single level 1 trauma centre. We also studied several physiological variables as they were monitored in the daily clinical practice and registered deviations from treatment goals based on guidelines for severe TBI. Because of the patient heterogeneity the cohort was divided into two groups depending on level of monitoring and care; patients staying in ICUs three days or more versus patients not staying or staying less than three days in ICUs.

## Methods

### Patients and setting

**Fig. 1:**
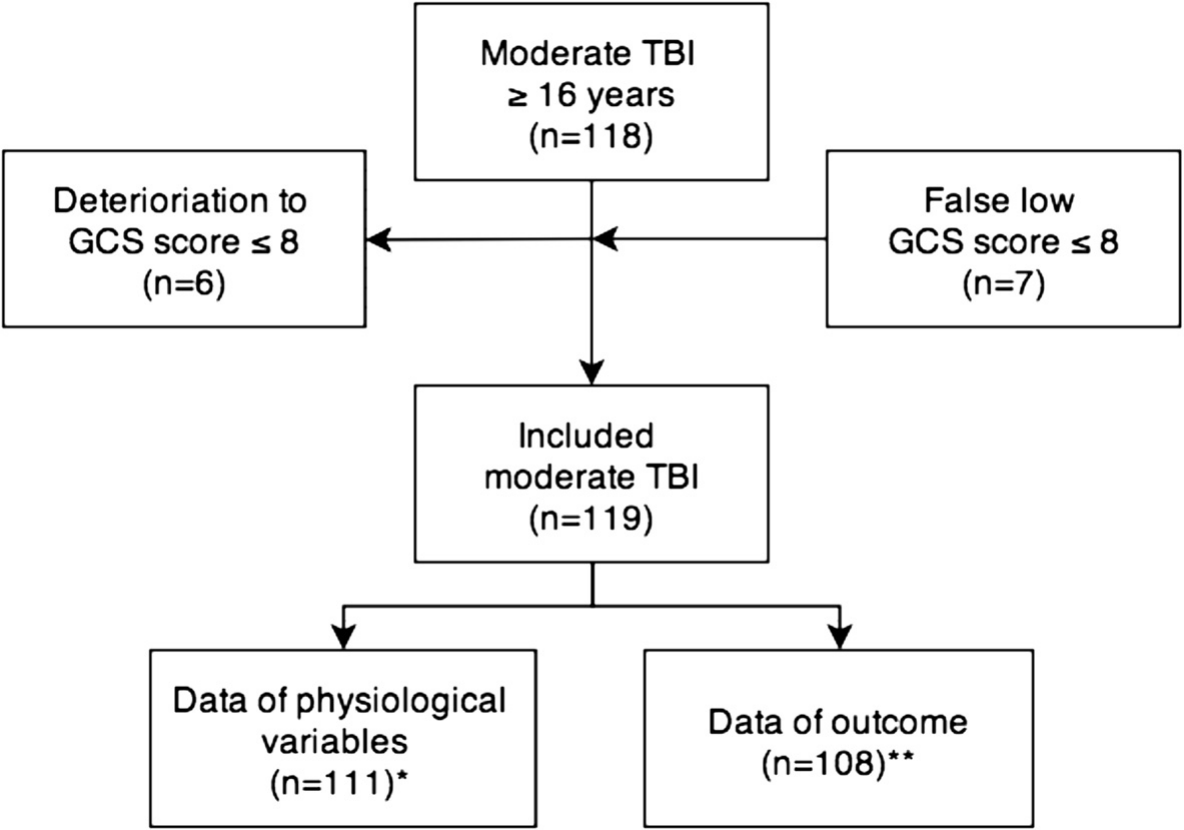
Flowchart of moderate TBI patients according to HISS (≥16 years) admitted to St. Olavs Hospital during a five-year period (2004–09). *Missing observation chart in 8 patients. ** Missing outcome for 11 patients due to: Death of other reasons than TBI (*n* = 5). Foreigners, not possible to reach (*n* = 1). Did not want to participate for follow-up (*n* = 4). Lost to follow-up (*n* = 1)

The study was conducted at St. Olavs Hospital, Trondheim University Hospital, a level 1 trauma centre and a tertiary referral centre for all neurosurgical activities in Mid-Norway, serving approximately 680 000 inhabitants. There are seven primary hospitals in the region, where some of the patients with moderate TBI may undergo diagnostic investigations and stabilization before transfer to the study hospital. Also an unknown number of patients with moderate TBI were managed at their primary hospital, without transfer to the study hospital. All patients with moderate TBI admitted to the study hospital were enrolled in a prospective longitudinal cohort study during a five-year period (October 2004 - October 2009). Injury-related data were collected by the resident on-call at department of neurosurgery who had contact with the primary hospitals or ambulance personnel. On admission to the study hospital, 118 patients ≥16 years were classified with moderate TBI according to the Head Injury Severity Scale (HISS): Glasgow Coma Scale (GCS) score 9–13, or GCS score 14–15 with loss of consciousness for five minutes or more and/or focal deficits [21]. Patient who deteriorated with GCS score decreasing to ≤ 8 after admission, were treated as severe TBI and were not included in this study(*n* = 6). Patients who were heavily intoxicated with admission GCS score ≤ 8, but who rapidly improved, were classified and treated as moderate TBI, and included in this study(*n* = 7). This decision was made during the study planning since the aim was to study the acute phase course and deviations in physiological variables of patients who had a truly moderate TBI [22, 23]. Hence, finally, 119 patients were included in the study (Fig. 1).

### Injury severity and acute phase course

Injury related variables were recorded prospectively. GCS score reported on admission to the study hospital was used if available (*n* = 81). If intubation was performed prior to admission to the study hospital, the pre-intubation GCS score at injury site (*n* = 15) or primary hospital (*n* = 16) was used. For the seven patients with obviously false low GCS score on admission due to high blood alcohol concentration, GCS scores were not used in the analysis (*n* = 7).

CT findings were reviewed by a radiologist (IHS) or a resident in radiology (KGM) in cooperation with neuroradiologists. Intracranial procedures included surgery for intracranial mass lesions (subdural, epidural or intracerebral) or depressed skull fracture and insertion of intracranial pressure monitoring devices (parenchymal intracranial pressure sensor and/or external ventricular drain) and other drains (cisternal or subdural).

Injury Severity Score (ISS) [24] was used as a measure for overall injury severity and the scoring was performed by residents in radiology (KGM) and neurosurgery (SH). Extracranial injuries included fractures (facial, extremity, pelvic or spinal), abdominal and thoracic injuries, major wounds or tissue lacerations. Extracranial surgery included surgery of these injuries, including insertion of thoracic drain, stitching of major wounds and lacerations.

During the study period (the first three days post-injury), for each 24 h, we registered if the patients stayed at home, in primary hospital or in the study hospital. Some patients were not admitted to the study hospital immediately after injury because of admission to primary hospital first, or they did not seek immediate medical assistance. Also some patients were discharged to their primary hospital or home before the study period ended. Hence, some patients were categorized as staying at home or at primary hospital some of the days during the study period. Patients who stayed in ICUs (general or neuro ICU) three days or more were defined as staying in ICUs ≥3 days while those staying at wards or in ICUs less than three days were defined as staying in ICUs 0–2 days.

### Monitoring of deviations in physiological variables

The selected physiological variables and the deviation limits were; systolic blood pressure; <90 mmHg, oxygen saturation (SatO_2_); <92 %/partial arterial pressure of oxygen (PaO_2_) <11 kPa, body temperature; ≥38° Celsius, serum haemoglobin; ≤10 g/dl, serum sodium; ≤135 mmol/l and glucose; ≥8 mmol/l. These were collected retrospectively from the medical records at the primary hospitals and the study hospital by the first author. There is lack of guidelines for treatment goals of physiological variables for moderate TBI. Hence, the deviation limits were set based on treatment goals for selected physiological variables in the guidelines used in severe TBI patients at the study hospital during the study period, based on international recommendations [18]. After the study period the treatment goal for glucose was set to ≤10 mmol/l at our hospital.

The study period for deviations in physiological variables was the first three days post-injury. If exact time of injury was unknown (*n* = 23), the observation period started at first documentation by medical personnel. If several deviations occurred in a time period of an hour, this was documented as only one deviation so the maximal number of possible deviations in one variable per day was 24.

In the ICUs, intra-arterial blood pressure and SatO_2_ were measured continuously and documented every hour. Blood samples were taken daily, or several times a day. The body temperature was measured continuously either by a rectal probe or via urinary catheter, or by intermittent measurements rectally, axillary or in-ear. The values measured externally were unadjusted according to recommendations in the literature [25]. At wards, non-invasive measurements of blood pressure, oxygen saturation and body temperature were performed on indication. Blood samples were usually taken every morning in the acute phase and as required. Since we evaluated deviations in physiological variables according to clinical practice, the number of measurements varied between the patients, and also between the two patient groups depending of the level of care. For some patients complete observation charts were not available for the first three days, and these were excluded from the deviation analyses (*n* = 8).

### Outcome

The brain injury related outcome was measured by a brain specific outcome measure; Glasgow Outcome Scale Extended (GOSE) [26], collected by telephone or face-to face 12 months post-injury by a specialist in physical medicine (TS), an occupational therapist or a research nurse. GOSE scores were dichotomized into GOSE scores 7–8 (without disability) and GOSE scores ≤6 (with disability).

### Statistical analysis

Descriptive data are presented as frequencies (percentages) and medians (range). Categorical variables were analysed using Pearsons Chi square or Fisher’s exact. Differences in means or medians between groups were analysed using *t*-test for independent samples or Mann -Whitney *U* test. A two sided *P-* value < 0.05 was regarded as statistically significant. Statistical analyses were performed using PASW version 19 (SPSS, Inc., IL, Chicago, USA).

### Ethical considerations

All patients with moderate TBI were enrolled in the database for the prospective longitudinal cohort study. Informed consent was obtained from the patients or their next of kin. The studies were conducted according to the Helsinki declaration and approved by the Regional Committee for Medical Research Ethics, Mid-Norway: 2004/protocol-number 135 and 2011/protocol-number 1804.

## Results

### Injury related and acute phase data

Median age for the 119 patients with moderate TBI was 47 years (range 16–92). Median admission GCS score was 12 (range 9–15), 86 % had intracranial pathology on CT and 61 % had extracranial injuries (Table 1).

**Fig. 2:**
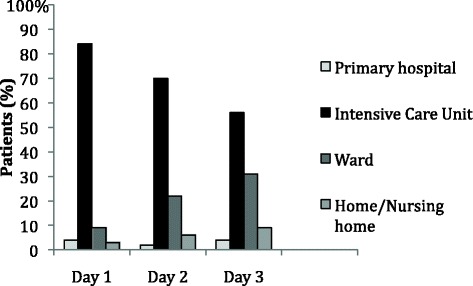
Moderate TBI patients stay day 1–3 post-injury

Thirty-one percent of the patients were transported via primary hospital. At admission to the study hospital, 32 patients (27 %) were intubated and 21 patients (18 %) needed mechanical ventilation during the entire study period (3 days). The first day after the injury 100 patients (84 %) stayed in ICUs (Fig. 2). 61 patients (51 %) stayed in ICUs ≥3 days and median stay in ICUs for these patients were 5 days (range 3–37 days).

**Table 1:** Demographic and injury-related data of all patients, patients staying in ICU ≥3 days and patients staying in ICU 0–2 days

Variable^a^	All patients *n* = 119	ICU ≥3 days *n* = 61 (51 %)	ICU 0–2 days *n* = 58 (49 %)	*P*-value^*^
Age (years)	47 (16–92)	50 (17–85)	43 (16–92)	0.210
Gender male	80 (67 %)	40 (66 %)	40 (69 %)	0.694
GCS score^b^	12 (9–15)	12 (9–15)	13 (9–15)	**0.001**
Injury mechanism				
Fall	54 (45 %)	25 (41 %)	29 (50 %)	0.323
RTA	47 (40 %)	31 (51 %)	16 (28 %)	**0.010**
Abnormal CT scan	107 (90 %)	58 (95 %)	49 (85 %)	0.055
Traumatic SAH	69 (58 %)	41 (67 %)	28 (48 %)	**0.036**
SDH	58 (49 %)	37 (61 %)	21 (36 %)	**0.008**
Multiple contusions	50 (42 %)	30 (49 %)	20 (35 %)	0.104
Skull fracture	55 (46 %)	34 (56 %)	21 (36 %)	**0.033**
Intracranial procedures				
Mass lesion or depressed skull fracture	19 (16 %)	9 (15 %)	10 (17 %)	0.803
ICP measurement	22 (18 %)	21 (34 %)	1 (2 %)	**<0.001**
ISS	20 (1–50)	24 (9–50)	16.5 (1–41)	**0.001**
Extracranial injury	72 (61 %)	47 (77 %)	25 (43 %)	**<0.001**
Extracranial surgery	38 (32 %)	23 (38 %)	15 (26 %)	0.166
GOSE score (≤6)^c^	41 (38 %)	28 (49 %)	13 (26 %)	**0.017**

^*^Comparison between patients staying in ICU ≥3 days and patients staying in ICU 0–2 days. Bold indicates *P*-values <0.05

^a^Values are reported as median (range) or n (%)

^b^Admission GCS scores were not available in seven of the patients due to drug/alcohol intoxication giving false low GCS score (*n* = 112). Pre-intubation GCS scores were used for patients intubated at injury site (*n* = 15) or at local hospital (*n* = 16)

^c^ *n* = 108

*ICU* intensive care unit, *GCS*, Glasgow Coma Scale, *RTA* road traffic accident, *CT* computer tomography, *SAH* subarachnoid haemorrhage or intraventricular haemorrhage, *SDH* subdural haematoma, *ICP* intracranial pressure, *ISS* injury severity score

Patients staying three days or more in ICUs (ICU ≥3 days) had lower median GCS score than those who did not stay or stayed less than three days in ICUs (ICU 0–2 days) (*P* = 0.001) (Table 1). They also had a higher frequency of traumatic subarachnoid haemorrhage (SAH) (*P* = 0.036), acute subdural haematoma (SDH) (*P* = 0.008), skull fractures (*P* = 0.033) and were more likely to have intracranial pressure (ICP) monitoring (*P* < 0.001). However, the two groups had the same frequency of surgery for intracranial mass lesions or depressed skull fractures. The patients staying in ICUs ≥3 days had a higher median ISS (*P* = 0.001) and more extracranial injuries (*P* < 0.001), and a higher frequency (20 % versus 3 %) of surgical interventions for chest injuries (*P* = 0.006).

For the entire cohort of 119 moderate TBI patients, 13 patients had admission GCS score 14–15 (with loss of consciousness ≥5 min and/or focal deficits). Their median age was 63 years (range 16–85). 12 patients (92 %) had intracranial pathology: 8 patients (62 %) had traumatic SAH, 8 patients (62 %) had SDH and 7 patients (54 %) had multiple contusions. Intracranial procedure due to mass lesion or depressed skull fracture was performed in 2 of the patients and 1 had an ICP device inserted. Median ISS was 13 (range 4–33), 7 patients (54 %) had extracranial injuries and 6 patients (46 %) had extracranial surgery. 3 patients (23 %) with admission GCS score 14–15 stayed in ICU ≥3 days, and only 1 patient had GOSE score ≤6 12 months post-injury.

### Deviations in physiological variables

Deviations were observed in both patients groups (Table 2). Patients staying in ICUs ≥3 days often had deviations from treatment goals in blood pressure, oxygen saturation, body temperature, haemoglobin and blood glucose (53-65 % of the patients).

**Table 2:** Patients (%) with deviations in the physiological variables

Variable (deviation limit)	ICU ≥3 days % (n/N^a^)	ICU 0–2 days % (n/N^a^)
Hypotension (BP < 90 mmHg systolic)	53 % (28/53)	35 % (19/54)
Hypoxia (PO2 < 11 kPa//SAT < 92 %)	57 % (31/54)	20 % (11/54)
Hyperthermia (Body tp ≥ 38° Celsius)	59 % (32/54)	33 % (18/54)
Anaemia (Hgb ≤ 10 g/dl)	56 % (30/54)	17 % (9/53)
Hyponatremia (Na ≤ 135 mmol/l)	19 % (10/54)	15 % (8/53)
Hyperglycaemia (Gluc ≥ 8 mmol/l)	65 % (35/54)	38 % (15/40)

^a^Lower numbers for some patients because physiological variable is not measured or observation chart not available/missing

*BP* blood pressure, *PO2* oxygen partial pressure, *SAT* oxygen saturation, *Body tp* body temperature, *Hgb* blood haemoglobin, *Na* serum sodium, *Gluc* blood glucose

The highest proportion of deviations related to number of measurements in patients staying in ICUs ≥3 days was observed for haemoglobin (33 %), followed by body temperature and blood glucose, 18 % and 19 % respectively (Table 3).

**Table 3:** Deviations related to total number of measurements (%) in the physiological variables

Variable (deviation limit)	ICU ≥3 days % (deviations/ measurements)	ICU 0–2 days % (deviations/ measurements)
Hypotension (BP < 90 mmHg systolic)	5 % (162/3216)	5 % (59/1131)
Hypoxia (PO2 < 11 kPa//SAT < 92 %)	4 % (127/3101)	2.5 % (27/1063)
Hyperthermia (Body tp ≥ 38° Celsius)	18 % (198/1070)	13 % (31/236)
Anaemia (Hgb ≤ 10 g/dl)	33 % (327/984)	17 % (42/250)
Hyponatremia (Na ≤ 135 mmol/l)	5 % (47/972)	5 % (13/246)
Hyperglycaemia (Gluc ≥ 8 mmol/l)	19 % (183/969)	18 % (39/216)

*BP* blood pressure, *PO2* oxygen partial pressure, *SAT* oxygen saturation, *Body tp* body temperature, *Hgb* blood haemoglobin, *Na* serum sodium, *Gluc* blood glucose

### Outcome

Patients staying in ICUs ≥3 days had worse outcomes (higher frequency of GOSE scores ≤6) one year post-injury than patients staying in ICUs 0–2 days (49 % versus 26 %, *P =* 0.017).

## Discussion

The present study showed that 84 % of all patients with moderate TBI admitted to a level 1 trauma centre were treated in ICUs during the acute phase, and half of them stayed three days or more in ICUs. As expected, these patients had more severe intracranial injuries but also more extracranial injuries than patients staying in ICUs 0–2 days. For most of the physiological variables, more than half of the patients staying in ICUs ≥3 days had at least one deviation from the treatment goals we use for patients with severe TBI. The proportion of measurements indicating anaemia was particularly high in this group.

### Injury severity and acute phase course

The percentage of patients with moderate TBI treated in ICUs in our study was high compared to other studies with moderate TBI patients. However, there are only a few comparable contemporary studies [3, 4, 7, 8], and to our knowledge none has presented a cohort of moderate TBI patients from a single level 1 trauma centre. In our study, 27 % were mechanically ventilated on admission, as many as 84 % were admitted to an ICU and half of them stayed there three days or more. In the study by Compagnone et al., in which 11 hospitals with neurosurgical units participated, 19 % of the patients with moderate TBI were intubated on admission [7]. In the multicentre study by Andriessen et al., including five level 1 trauma centres, 36 % were mechanically ventilated at admission and 43 % were treated in ICUs [8]. Only 20 % were treated in ICUs in the study by Fabrri et al., however, this study was conducted at a general hospital without neurosurgical service [4]. In the study by Vitaz et al., 96 % of the patients required mechanical ventilation, but this cohort may not be comparable to ours due to differences in patient population and acute phase management [3].

Our study had a high proportion of patients with intracranial pathology on CT (86 %) compared to some of the above mentioned studies reporting 56-65 % [4, 7, 8]. Other studies also report lower median ISS [4, 8], and lower frequency of extracranial injuries (21-24 %) [7, 8]. This could be explained by the fact that one third of our patients were secondary referrals from primary hospitals, and most major traumas, typically road traffic accidents (RTAs), are admitted to the regional trauma centre. We also know that some patients not needing neurosurgical intervention stay at their primary hospital for observation and treatment in the acute phase. Hence, our study did not capture all patients with moderate TBI in the whole region. However, the study by Andriessen et al. from a level 1 trauma centre had the same percentage of RTA, but still lower frequencies of intracranial CT findings (56 %) and extracranial injuries (24 %) [8]. Therefore, it seems difficult to compare different cohorts of patients with moderate TBI.

We found that the cohort of moderate TBI had two different clinical pathways in the acute phase as half of them stayed in ICU three days or more, and the other half stayed in ICUs 0–2 days. Median GCS score in the group staying in ICUs ≥3 days was significantly lower than in the patients staying in ICUs 0–2 days. Although the difference was only one point at the GCS, this might indicate more severe intracranial injuries and need for closer observation. This would have been even more pronounced if the 6 patients with moderate TBI who deteriorated to severe TBI rapid after admission had been included in the analyses. The two groups had the same frequency of surgery for mass lesions or depressed skull fractures, implicating that also some patients needing neurosurgery had ICU stays of less than three days. Conversely, some of the patients with minor intracranial injuries as detected by CT, may need extra observation and ICU stays because of disorientation and cognitive deficits [27, 28]. Studies of clinical MRI have shown that many patient with moderate TBI have lesions of traumatic axonal injury despite no or minor CT findings, which might explain their cognitive deficits [29]. Patients staying in ICUs ≥3 days also had more extracranial injuries, including chest injuries requiring thoracic drainage. This was also evidenced by significantly higher ISS.

The combination of intra- and extracranial injuries can be one reason for the high proportion of patients admitted to ICUs in the acute phase in our study. When comparing ICU admissions across different centres, however, other factors, such as patient characteristics, routines for intubation and importantly, available resources may also influence whether patients with moderate TBI are treated in ICUs in the acute phase.

Compagnone et al. also divided their cohort of moderate TBI patients into two groups based on differences in acute phase course and outcome prediction [7]. They found more extracranial injuries in the group with the lowest GCS score. Our study also showed that more patients staying in ICUs ≥3 days had worse outcomes (higher frequency of GOSE scores ≤6) than patients staying in ICUs 0–2 days 12 months post-injury. This was as expected and corresponded to the fact that the TBI was more severe with lower GCS scores in the first group.

### Deviations in physiological variables

No other study has reported physiological variables for moderate TBI patients separately during more than the first hours or day post-injury [6, 30]. Most of the patients staying in ICUs ≥3 days had comprehensive documentation of physiological variables during the entire study period. Therefore we find it more applicable to discuss the implications of deviations in physiological variables for this group.

At least one deviation in blood pressure and oxygen saturation was seen in more than half of the patients staying in ICUs ≥3 days. Blood loss due to extracranial injuries and surgery, or administration of analgesics and sedatives are all possibly reasons for hypotensive episodes. Chest injuries, lung contusions and the need for thoracic drainage and mechanical ventilation might explain most of the hypoxic episodes. Also, lower GCS scores may affect optimal oxygenation in patients that are not mechanically ventilated. However, the proportion of deviations related to the total number of measurements was low for both blood pressure and oxygenation. This finding suggests that these episodes were short in duration and effectively corrected, which is in accordance with other studies showing that ensuring adequate circulation and ventilation is a priority for ICU- nurses when caring for critically ill TBI patients [6, 31].

We also found that patients staying in ICUs ≥3 days often had anaemia, which might be related to the high incidence of extracranial injuries. It may also be related to haemodilution resulting from excessive fluid administration. However, it seems that anaemia is not routinely corrected in patients with moderate TBI, due to the high number of deviations in this variable. A reason can be the lack of agreement in recommendations for blood transfusion for both moderate and severe TBI patients [32, 33].

Hyperthermia and hyperglycaemia were frequently seen in the patients staying in ICUs ≥3 days. These findings correspond well with the normal stress response after trauma [13, 15, 34, 35], and can be related to both the intra- and extracranial injuries. An increased focus on the best methods for fever reduction in patients with TBI has been suggested [35].

The frequencies and proportions of deviations will differ due to the nature of the different physiological variables. However, lack of guidelines for monitoring and treating deviations in these variables may leave them unnoticed and uncorrected. Recently multimodal monitoring toward targeted therapy for severe TBI has been recommended. Such individualization of management can optimize recovery and also prevent overtreatment [36]. This is highly relevant for moderate TBI patients, as our study confirms they are a heterogeneous group with great variability in clinical course. In addition, monitoring and treatment of deviations in physiological variables in patients with moderate TBI might be challenging due to disorientation, cognitive deficits and lack of cooperation in some of the patients.

### Classification of moderate TBI in this study

We reclassified six patients who rapidly deteriorated after admission and seven patients with falsely low GCS score due to intoxication. This was done since the aim was to study the acute phase course and deviations in physiological variables of patients that had a truly moderate TBI. Since we used the HISS classification, patients with GCS score 13, and also 14–15 with loss of consciousness ≥ 5 min and/or focal deficits, were included in this study. However, these latter patients are often categorized into the mild TBI- group, and if they have intracranial findings, as mild complicated TBI [37]. The TBI patients with GCS score 14–15 in our study had high median age, most of them had intracranial pathology on CT scan, and therefore they did not seem to have less severe intracranial injuries. Many clinicians and researchers question the validity of GCS score for TBI classification, suggesting multimodal classifications system combining different indicators for severity classification [1]. We believe that the present study supports this view.

### Strengths and limitations

Our study included all patients with moderate TBI ≥ 16 years admitted to a level 1 trauma centre, serving seven primary hospital covering the middle region of Norway. The study period and the single centre design allowed for a limited sample size, and an unknown number of patients with moderate TBI were managed at their primary hospital, without referral to the study hospital. Our study had an observational design with prospective registration of most variables. However, the values of the physiological variables were registered retrospectively and measured as they were observed in daily clinical practice. Hence, we were not able to record duration and severity of deviations, and also documentation of physiological variables were lacking in many patients, especially in those staying at wards. In addition, we do not know if there were any individualized target values for the physiological variables for patients with other concurrent diseases.

## Conclusion

This observational cohort study from a Norwegian single level 1 trauma centre, shows that the vast majority of the patients with moderate TBI were admitted to ICUs, half of them for at least three days due to intracranial but also extracranial injuries. More than half of the patients staying in ICU ≥3 days had at least one deviation from treatment goals in blood pressure, oxygen saturation, body temperature, haemoglobin and blood glucose. Also the proportion of measurements indicating anaemia was high in this group. Although many of these deviations are a natural response to trauma, we believe that lack of guidelines for monitoring and treatment in the acute phase allow for many of these deviations to pass uncorrected.

Seemingly, moderate TBI is a neglected group both in research and in the clinics. The ongoing large scale EU study CENTER-TBI, addresses the issue of level of care for TBI patients. Based on our results, we suggest that in research on patients with moderate TBI a differentiation of patients, with regard to the need for monitoring and level of care the first days post-injury, will contribute to the improvement of acute phase management.
